# 存在驱动基因突变肺鳞癌患者的临床病理特征及预后分析

**DOI:** 10.3779/j.issn.1009-3419.2016.10.02

**Published:** 2016-10-20

**Authors:** 童童 张, 峻岭 李

**Affiliations:** 100021 北京，国家癌症中心/中国医学科学院北京协和医学院肿瘤医院 National Cancer Center/Cancer Hospital, Chinese Academy of Medical Sciences and Peking Union Medical College, Beijing 100021, China

**Keywords:** 肺鳞癌, 表皮生长因子受体, 间变淋巴瘤激酶, *KRAS*基因, Lung squamous cell carcinoma, Epidermal growth factor receptor, Anaplastic lymphoma kinase, *KRAS* gene

## Abstract

**背景与目的:**

表皮生长因子受体（epidermal growth factor receptor, EGFR）、间变淋巴瘤激酶（anaplastic lymphoma kinase, ALK）以及*KRAS*基因是非小细胞肺癌（non-small cell lung cancer, NSCLC）常见的驱动基因。多项临床研究已证实，*EGFR*敏感突变及棘皮动物微管相关类蛋白4-间变淋巴瘤激酶（echinoderm microtubule-associated protein like 4-anaplastic lymphoma kinase, *EML4*-*ALK*）基因重排的晚期肺腺癌患者，一线治疗选择靶向药物优于化疗，其在总缓解率（overall response rate, ORR）、无进展生存期（progression-free survival, PFS）及生活质量方面均有明显优势。然而，在肺鳞癌患者中，目前尚无明确的基因检测位点及靶向药物来指导临床治疗。本研究旨在分析肺鳞癌患者*EGFR*、*ALK*以及*KRAS*基因的突变状态，以及相关的临床病理特征，从而为今后的治疗提供相应指导。

**方法:**

回顾性分析已行相关驱动基因检测的肺鳞癌患者90例，利用突变扩增阻滞系统（amplification refractory mutation system, ARMS）的方法进行*EGFR*及*KRAS*基因检测，荧光原位杂交（fluorescence *in situ* hybridization, FISH）技术进行*ALK*基因融合检测。

**结果:**

90例患者均进行了*EGFR*及*KRAS*基因检测，8例患者为*EGFR*基因突变（8.8%）；2例患者为*KRAS*基因突变（2.2%）；18例患者通过FISH方法进行了*ALK*基因融合检测，1例患者存在*EML4*-*ALK*基因融合（5.6%）。女性患者的*EGFR*基因突变率高于男性（*P*=0.022）。*EGFR*突变及野生型患者在病理分期（*P*=0.042）及分化程度（*P*=0.003）上均有差异。以胸膜为首发转移部位的*EGFR*突变患者比率高于*EGFR*野生型患者（*P*=0.013）。靶向治疗与化疗对*EGFR*突变患者的PFS无影响（*P*=0.607）。

**结论:**

*ALK*基因融合患者应用靶向治疗后可获得理想疗效。

## Abstract (en)

**Background and objective:**

It has been proven that epidermal growth factor receptor (*EGFR*), anaplastic lymphoma kinase (*ALK*), and *KRAS* are common driver genes in non-small cell lung cancer (NSCLC). Molecular targeted therapy increases the overall response rate and progression-free survival (PFS) of patients with EGFR-sensitive mutation or echinoderm microtubule-associated protein like 4-anaplastic lymphoma kinase (*EML4*-*ALK*) fusion. However, target and targeted drugs for lung squamous cell carcinoma to indicate clinical therapy remain to be confirmed. The aim of this study was to analyze the relationship between the status of driver genes and the clinicopathologic characteristics of NSCLC.

**Methods:**

A total of 90 patients were recruited and tested for *EGFR*, *ALK* and *KRAS* mutations. The status of *EGFR* and *KRAS* was tested by amplification refractory mutation system, and the status of ALK was tested by fluorescence *in situ* hybridization.

**Results:**

Of the 90 patients, 8 patients had *EGFR* mutation (8.8%), and 2 cases had *KRAS* mutation (2.2%). *EML4*-*ALK* fusion was found in 1 of 18 patients (5.6%). *EGFR* mutation occurred more frequently in females than in males (*P*=0.022). Significant differences were observed in pathological stage (*P*=0.042) and differentiation grade (*P*=0.003). No significant difference in PFS was observed between EGFR-TKI treatment and chemotherapy in *EGFR* mutation patients with squamous cell carcinoma of the lung (*P*=0.607). Patients with *EML4*-*ALK* fusion could benefit from targeted therapy.

**Conclusion:**

*EML4*-*ALK* fusion occurred more frequently than *EGFR* and *KRAS* mutations in patients with lung squamous cell carcinoma. Clinicopathologic characteristics were different between *EGFR* mutation and *EGFR* wild-type patients. The relationship between molecular targeted therapy and status of *EGFR* or *ALK* genes in patients with lung squamous cell carcinoma needs further investigation.

目前，肺癌是引起肿瘤死亡的主要原因^[1]^。据统计，新确诊的肺癌患者中，非小细胞肺癌（non-small cell lung cancer, NSCLC）约占85%，其中最常见的两大病理类型为腺癌和鳞癌，分别占50%和30%^[2]^。靶向药物上市之前，晚期NSCLC患者的治疗策略为以铂类药物为基础的联合化疗。与最佳支持治疗相比，尽管联合化疗可以延长患者的总生存期（overall survival, OS）^[3]^，但其有效率仅为20%左右，中位生存也只有8个月-10个月^[4]^。近年来，随着对NSCLC相关基因改变的深入研究，一系列与NSCLC相关的基因已被发现，这些诱发和维持恶性肿瘤发生发展的分子改变被称为驱动基因。这些驱动基因编码的蛋白与细胞的增殖与生存密切相关。驱动基因这一概念认为肿瘤的发生以某一特定基因的表达为基础，诱发和维持肿瘤的发生发展^[5]^。

多项临床研究已证实，表皮生长因子受体（epidermal growth factor receptor, *EGFR*）敏感突变及棘皮动物微管相关类蛋白4-间变淋巴瘤激酶（echinoderm microtubule-associated protein like 4-anaplastic lymphoma kinase, *EML4*-*ALK*）基因重排的晚期肺腺癌患者，一线治疗选择靶向药物优于化疗，其在总缓解率（overall response rate, ORR）、无进展生存期（progression-free survival, PFS）及生活质量方面均有明显优势^[6-17]^。然而，在肺鳞癌患者中，目前尚无明确的基因检测位点及靶向药物来指导临床治疗^[18]^。靶向药物是否同样能给肺鳞癌患者带来巨大的临床获益，目前仍缺乏大规模前瞻性临床研究数据。本研究通过回顾性分析肺鳞癌基因检测患者的临床病理特征，以期为今后的临床治疗提供依据。

## 1 资料与方法

### 1.1 资料

筛选中国医学科学院肿瘤医院2011年1月-2014年12月收治的肺鳞癌患者2, 858例，回顾性分析了其中90例已行基因检测的患者，所有患者均经病理组织学免疫组化证实为肺鳞癌。其中男性79例，女性11例。年龄32岁-73岁；按国际肺癌研究协会（International Association for the Study of Lung Cancer, IASLC）2009年第7版NSCLC的分期标准，Ⅰ期24例，Ⅱ期19例，Ⅲ期37例，Ⅳ期10例。

### 1.2 基因检测方法

*EGFR*基因突变应用突变扩增阻滞系统（amplification refractory mutation system, ARMS）的方法，*KRAS*基因突变也应用ARMS法检测，*ALK*基因融合应用荧光原位杂交（fluorescence *in situ* hybridization, FISH）技术进行检测。

### 1.3 统计学方法

采用SPSS 22.0统计软件进行统计分析，基因突变状态和临床特征分析采用卡方检验和*Fisher’s*精确检验，生存分析采用*Kaplan*-*Meier*生存曲线。以*P*＞0.05为差异有统计学意义。

## 2 结果

### 2.1 突变检测结果

2, 858例肺鳞癌患者中，90例患者进行了相关驱动基因检测，检测率仅为3.1%。其中所有患者均同时检测了*EGFR*及*KRAS*基因，8例患者为*EGFR*基因突变（4例患者突变位点为19号外显子缺失，4例患者突变位点不详），突变率为8.8%；2例患者为*KRAS*基因突变，突变位点均为12号密码子，突变率为2.2%；18例患者通过FISH方法进行了*ALK*基因融合检测，1例患者存在*EML4*-*ALK*基因融合，阳性率为5.6%（表 1）。

**1:** *EGFR*、*KRAS*和*ALK*基因突变检测结果 Results of *EGFR*, *KRAS* and *ALK* mutation status

Item	Sample number	Percent
*EGFR* gene	8	
Exon 19 (19del)	4	4.4%
Unknown	4	4.4%
*KRAS* gene	2	
Codon 2	2	2.2%
*ALK* gene	1	
*EML4*-*ALK*	1	5.6%
EGFR: epidermal growth factor receptor; ALK: anaplastic lymphoma kinase; EML4-ALK: echinoderm microtubule-associated protein like 4-anaplastic lymphoma kinase.		

### 2.2 *EGFR*基因突变与临床特征的关系

女性患者的*EGFR*基因突变率高于男性（3/11 *vs* 5/79），差异有统计学意义（*P*=0.022）。*EGFR*突变患者低、中分化比率低于*EGFR*野生型患者（62.5% *vs* 93.9%, *P*=0.003），*EGFR*基因突变患者确诊时多处于Ⅲ期-Ⅳ期（87.5% *vs* 48.7%, *P*=0.042）。以胸膜为首发转移部位的*EGFR*突变患者比率高于*EGFR*野生型患者（37.5% *vs* 8.5%, *P*=0.013）。其他首发转移部位在*EGFR*突变及野生型患者中的发生率无统计学差异（*P*＞0.05）（表 2）。

**2:** *EGFR*基因突变与临床特征的关系（*n*=90） *EGFR* gene mutation and clinical characteristic relationship (*n*=90)

Clinical characteristic	Data	*EGFR* gene		*P*
		Mutation (*n*=8)	Wild type (*n*=82)	
Gender				0.022
Female	11 (12.2%)	3 (37.5%)	8 (9.8%)	
Male	79 (87.8%)	5 (62.5%)	74 (90.2%)	
TNM stage				0.042
Ⅰ	24 (26.7%)	1 (12.5%)	23 (28.0%)	
Ⅱ	19 (21.1%)	0 (0)	19 (23.2%)	
Ⅲ	37 (41.1%)	4 (50.0%)	33 (40.2%)	
Ⅳ	10 (11.1%)	3 (37.5%)	7 (8.5%)	
Pathological grade				0.003
Low	44 (48.9%)	2 (25.0%)	42 (51.2%)	
Moderate	38 (42.2%)	3 (37.5%)	35 (42.7%)	
High	2 (2.2%)	0 (0)	2 (2.4%)	
Unknown	6 (6.7%)	3 (37.5%)	3 (3.7%)	
Metastatic site				
Lung	9 (10.0%)	2 (25.0%)	7 (8.5%)	0.138
Bone	7 (7.8%)	0 (0)	7 (8.5%)	0.389
Brain	6 (6.7%)	1 (12.5%)	5 (6.1%)	0.488
Pleura	10 (11.1%)	3 (37.5%)	7 (8.5%)	0.013
Liver	4 (4.4%)	0 (0)	4 (4.9%)	0.523
TNM: tumor-node-metastasis.				

### 2.3 基因突变与靶向治疗疗效

8例*EGFR*敏感突变的患者中，4例患者应用EGFR-TKIs靶向治疗，4例患者应用化疗。8例*EGFR*突变患者的中位PFS为5.3个月，应用靶向治疗及化疗对PFS影响无统计学差异，分别为5.3个月和5.4个月（*P*=0.607）。1例*ALK*基因融合患者应用克唑替尼靶向治疗，最佳疗效评价为部分缓解（partial response, PR），PFS达到10个月。

## 3 讨论

目前，肺鳞癌患者是否需要同肺腺癌患者那样常规进行驱动基因检测仍存在一定争议。美国临床肿瘤学会（American Society of Clinical Oncology, ASCO）认为，所有NSCLC患者均应一线进行*EGFR*基因检测从而指导后续治疗^[19]^。欧洲肿瘤内科学会（European Society for Medical Oncology, ESMO）建议在非鳞癌的NSCLC患者中进行驱动基因检测^[20]^。美国国立综合癌症网站（National Comprehensive Cancer Network, NCCN）则建议对肺鳞癌，尤其是不吸烟、小活检或存在混合成分的鳞癌患者也应进行*EGFR*基因检测^[21]^。这些争议存在的原因是由于目前没有有力证据证明EGFR-TKIs能给肺鳞癌患者带来明显的临床及生存获益。

对于常见驱动基因（*EGFR*、*KRAS*及*ALK*）突变的发生率也仍存在一定争议。一项*meta*分析^[22]^显示，东亚人群中肺鳞癌*EGFR*的突变率略高于西欧人群，分别为4.6%和3.3%。而*KRAS*突变及*EML4*-*ALK*基因融合在两种族的肺鳞癌患者中却有明显差异，西欧人群的发生率均明显高于东亚人群（6.4% *vs* 1.8%, 4.5% *vs* 1.8%）。而国内衣素琴等^[23]^在48例肺鳞癌中检测到12例*EGFR*基因突变，突变率为25%。本研究结果中*EGFR*突变率为8.9%，介于国外报道及国内报道数据之间。*KRAS*突变率为2.2%，与国外研究数据相似。而*EML4*-*ALK*融合发生率在本研究中可达5.6%，明显高于国外报道，这可能与本研究进行*ALK*基因融合检测的样本量较少有关。然而，本研究数据证明至少在肺鳞癌患者中存在常见的驱动基因，且女性*EGFR*突变率明显高于男性，但无统计学差异。国内张卉等^[24]^进行的研究数据显示，*EGFR*突变与分期无关。而在本研究中，*EGFR*突变患者病理分期多处于Ⅲ期-Ⅳ期，且病理多为低-中分化。但由于样本量过小，仍需大样本数据证实。

*EGFR*敏感突变的肺腺癌患者，在接受EGFR-TKIs靶向治疗后，OS可延长至30个月。然而，目前EGFR-TKIs药物是否能给肺鳞癌患者带来生存获益仍缺乏有力证据。一项回顾性研究^[25]^数据显示，EGFR-TKIs治疗可使肺鳞癌患者生存得到延长，然而这些患者中大部分*EGFR*基因突变状态不明确。OPTIMAL研究^[26]^数据显示，在不明确病理类型的情况下，厄洛替尼可使所有*EGFR*敏感突变患者的生存得到延长。我们的研究数据显示，与传统化疗相比，*EGFR*突变的肺鳞癌患者在应用靶向治疗后PFS未能得到明显延长。这可能与样本量较小，且一部分患者并未在一线治疗时选择靶向药物有关。然而，由于靶向药物的安全性好、患者依从性较高，这类药物仍可作为*EGFR*突变肺鳞癌患者的一个有效治疗选择。

本研究结果显示，*ALK*基因融合的肺鳞癌患者在应用靶向治疗后PFS得到明显延长。然而由于本研究中仅有1例*ALK*基因融合患者，且目前对于靶向治疗在*ALK*基因融合肺鳞癌患者中的研究数据较少，尚不能明确靶向药物在*ALK*基因融合肺鳞癌患者中的治疗地位，仍需进一步的前瞻性研究加以探索。
